# Mitochondrial DNA Instability in Mammalian Cells

**DOI:** 10.1089/ars.2021.0091

**Published:** 2022-05-06

**Authors:** Gustavo Carvalho, Bruno Marçal Repolês, Isabela Mendes, Paulina H. Wanrooij

**Affiliations:** Department of Medical Biochemistry and Biophysics, Umeå University, Umeå, Sweden.

**Keywords:** mitochondrial DNA, genome instability, DNA replication

## Abstract

**Significance:**

The small, multicopy mitochondrial genome (mitochondrial DNA [mtDNA]) is essential for efficient energy production, as alterations in its coding information or a decrease in its copy number disrupt mitochondrial ATP synthesis. However, the mitochondrial replication machinery encounters numerous challenges that may limit its ability to duplicate this important genome and that jeopardize mtDNA stability, including various lesions in the DNA template, topological stress, and an insufficient nucleotide supply.

**Recent Advances:**

An ever-growing array of DNA repair or maintenance factors are being reported to localize to the mitochondria. We review current knowledge regarding the mitochondrial factors that may contribute to the tolerance or repair of various types of changes in the mitochondrial genome, such as base damage, incorporated ribonucleotides, and strand breaks. We also discuss the newly discovered link between mtDNA instability and activation of the innate immune response.

**Critical Issues:**

By which mechanisms do mitochondria respond to challenges that threaten mtDNA maintenance? What types of mtDNA damage are repaired, and when are the affected molecules degraded instead? And, finally, which forms of mtDNA instability trigger an immune response, and how?

**Future Directions:**

Further work is required to understand the contribution of the DNA repair and damage-tolerance factors present in the mitochondrial compartment, as well as the balance between mtDNA repair and degradation. Finally, efforts to understand the events underlying mtDNA release into the cytosol are warranted. Pursuing these and many related avenues can improve our understanding of what goes wrong in mitochondrial disease. *Antioxid. Redox Signal*. 36, 885–905.

## Introduction

Mitochondria are organelles that are well known for their remarkable efficiency in energy production. They contain their own genome, which in humans is organized as a circular double-stranded DNA (dsDNA) molecule of 16,568 bp, with genes encoding 22 tRNAs, 2 rRNAs, and 13 protein subunits of the electron transport chain (ETC) involved in oxidative phosphorylation (OXPHOS). Mitochondrial DNA (mtDNA) is present in hundreds or thousands of copies per cell, depending on the cell type (28). The coding information it contains is essential for efficient energy production, and hence its loss can lead to a variety of human diseases (176). Because of the multicopy nature of mtDNA, a cell can contain more than one type of mtDNA genome, a state referred to as heteroplasmy. The level of heteroplasmy—the fraction of mtDNA molecules carrying a specific mutation or deletion—can determine the progression of mitochondrial pathologies caused by mutations in mtDNA (176).

The two strands of mtDNA are annotated as heavy (H-) and light (L-) based on their differential separation on cesium chloride gradients. The mitochondrial genome is extremely information-dense, with little non-coding DNA other than the *ca* 1100 bp control region that contains the origin of Heavy-strand replication (O_H_) and the promoters for transcription of both strands. This region usually contains a hybridized third DNA strand, the 7S DNA, giving rise to the characteristic D-loop structure. The origin of Light-strand replication (O_L_) is located *ca* two-thirds downstream of O_H_. The specifics of mtDNA organization, maintenance, and expression have been recently reviewed (28, 46).

**FIG. 1.**
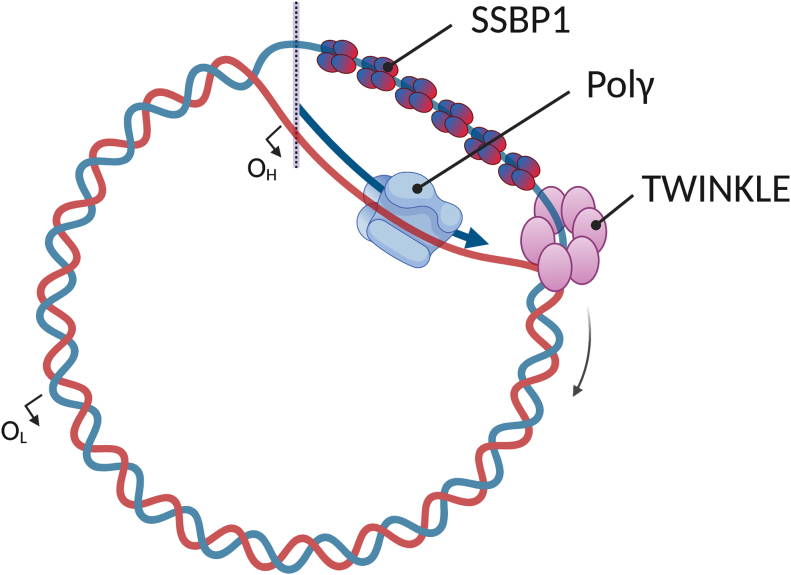
**Essential components of the mtDNA replication machinery.** According to the strand-displacement model, mtDNA replication starts at the O_H_. The replication fork proceeds continuously and unidirectionally in the direction indicated by the *curved arrow*, displacing the opposite strand. When the replication fork reaches the O_L_, the synthesis of the new L-strand is initiated. The mitochondrial RNA polymerase (not shown) synthesizes primers at both origins, and they are extended by the replicative Polγ. TWINKLE unwinds the mtDNA double-helix, and the displaced single-stranded mtDNA is stabilized by the homotetrameric SSBP1 protein. For a review of the different mtDNA replication models, please see Pohjoismäki *et al*. (126). mtDNA, mitochondrial DNA; O_H_, origin of Heavy-strand replication; O_L_, origin of Light-strand replication; Polγ, polymerase γ. Color images are available online.

The duplication of mtDNA is carried out by a set of proteins that is distinct from the machinery that replicates the nuclear genome (Fig. 1). The mitochondrial replicative DNA polymerase γ (Polγ) is a heterotrimer comprising one PolγA catalytic subunit and two PolγB accessory subunits. The catalytic subunit possesses DNA polymerase and 3′-5′ exonuclease (proofreading) activities, in addition to 5′-deoxyribose phosphate (dRP) lyase activity, and its processivity is increased by the presence of PolγB in the holoenzyme (46). To replicate dsDNA, Polγ requires the DNA unwinding activity of the hexameric TWINKLE helicase (77). The activity of both Polγ and TWINKLE is further stimulated by the mitochondrial single-stranded DNA-binding-protein SSBP1 that, along with the other two components of the core replisome, is essential for mtDNA replication (35). In addition to these proteins, the successful duplication of mtDNA involves other factors, including the mitochondrial RNA polymerase POLRMT that synthesizes the replication primers, ribonuclease H1, topoisomerases, and the MGME1 nuclease, to name a few (35).

In general, diverse factors can affect replication fidelity and prevent the normal progression of the mitochondrial replisome. Such replication stress can arise from chemically modified (*e.g*., oxidized, alkylated, or deaminated) nucleotides, incorporated ribonucleotides (rNMP, ribonucleoside monophosphate), secondary structures (*e.g*., G-quadruplexes), ultraviolet (UV)-light induced lesions, and topological stress in the template, as well as from an insufficient or unbalanced nucleotide pool (34, 69, 111, 170, 181). The mitochondrial replisome can also be challenged by pathological mutations in its components (33, 173). Mutations in Polγ and TWINKLE have been identified as causative of progressive external ophthalmoplegia (PEO), a late-onset mitochondrial disorder associated with mtDNA depletion and/or mtDNA mutations and deletions (33, 173). Mutations in these central enzymes are also found in a broad spectrum of other disease states, including mtDNA depletion syndrome (MDS), Alpers syndrome, Kearns-Sayre syndrome, ataxia-neuropathy, male infertility, and Perrault syndrome (21, 124). Recent studies revealed mutations in SSBP1 associated with optic atrophy, Pearson, Leigh, and Kearns-Sayre syndromes, resulting in mtDNA deletion or depletion (29, 45, 61, 125). In addition to mitochondrial diseases, mtDNA instability can contribute to various outcomes such as aging, cancer, and neurodegenerative disease (162, 175, 176).

**FIG. 2.**
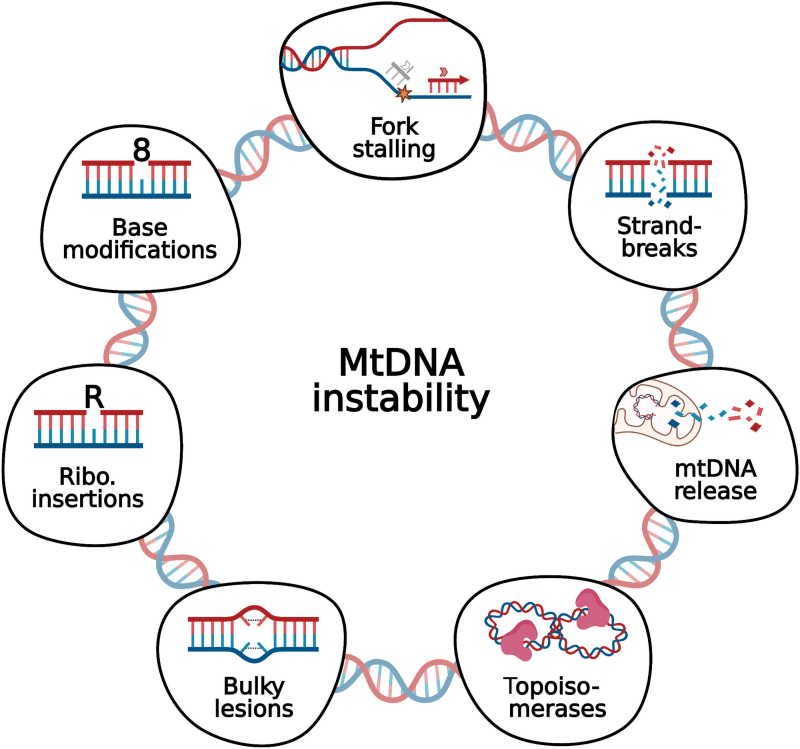
**Multiple sources of mtDNA instability.** Some of the many factors affecting mtDNA integrity that are discussed in this review range from small base modifications (*e.g*., 8-oxodG) and ribonucleotide insertions to bulky lesions such as UV-induced pyrimidine dimers. Persistence of lesions in the mtDNA, as well as the presence of secondary structures (*e.g*., G-quadruplexes) and unbalanced nucleotide supply, can potentially decrease the speed of the replication and cause fork stalling, possibly leading to DNA breaks. DNA DSBs are a severe form of damage to our genomes, and much has been debated as to whether broken mtDNA molecules can be repaired or not in the mitochondria. Recently, an increasing number of publications has paid attention to the cellular immune response on mtDNA release into the cytosol. Finally, the replication and transcription of mtDNA generates topological tensions that need to be alleviated by efficient action of topoisomerases. 8-oxodG, 8-oxo-2′-deoxyguanosine; DSB, double-strand break; UV, ultraviolet. Color images are available online.

In this review, we will discuss features that challenge the progression of the mitochondrial replisome, leading to mtDNA instability (Fig. 2). We also cover strategies for coping with this replicative stress, that is, additional polymerases and DNA repair factors that localize to mitochondria. Finally, we briefly review an emerging topic related to cellular responses to mtDNA instability, the activation of the innate response by cytosolic mtDNA. Deletions and point mutations, as well as defects in the replication machinery have been recently reviewed by others (33, 35, 117).

## Additional Polymerases Safeguard the Mitochondrial Genome

**FIG. 3.**
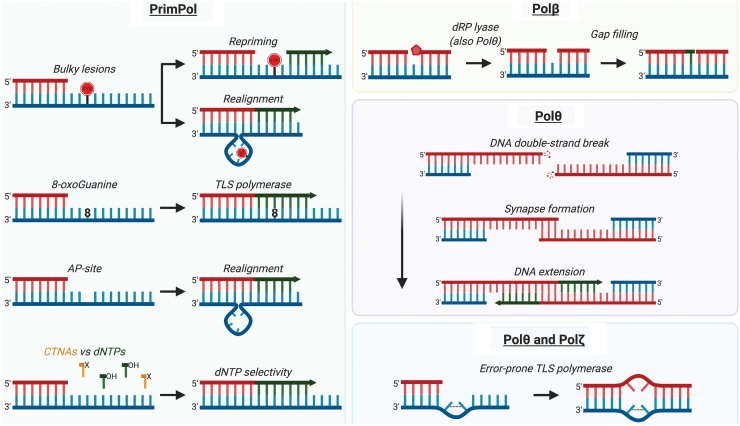
**Additional polymerases in the mitochondria.** Apart from the replicative Polγ, mitochondria also contain TLS polymerases to assist mtDNA replication and repair processes. The biochemical activities performed by these polymerases are depicted, but whether some activities occur in the mitochondria *in vivo* still lacks evidence. Primpol is primarily believed to rescue stalled replication forks by repriming ahead of the damage (*e.g*., UV-lesions, G-quadruplexes, blocking agents). PrimPol is a versatile DNA primase-polymerase that can bypass small lesions such as 8-oxodG by acting as a TLS polymerase or, alternatively, performing primer/template realignment based in sequence microhomology past “unreadable” damage (*e.g*., AP-sites, bulky lesions). It also presents higher discrimination than Polγ against CTNAs. Polβ, such as Polθ, can perform DNA-end processing through its 5′-dRP lyase activity. Polβ is a specialized gap-filling polymerase, acting mainly in BER. In the nuclei, Polθ is implicated in the repair of DNA DSBs mediated by sequence microhomology (MMEJ), although a role in mitochondrial DSB repair remains to be demonstrated. Polθ promotes DNA synapse formation between single-strand DNA overhangs, followed by DNA extension. Polθ and Polζ are known error-prone TLS polymerases that are able to bypass more mutagenic lesions (*e.g*., AP-sites, thymine glycol, UV-lesions) by inserting nucleotides opposite them or extending a lesion-containing mismatch. AP, apurinic or apyrimidinic; BER, base excision repair; CTNA, chain-terminating nucleoside analog; dRP, deoxyribose phosphate; MMEJ, microhomology-mediated end joining; TLS, translesion synthesis. Color images are available online.

Template damage that cannot be tolerated by replicative DNA polymerases may be bypassed with the help of translesion synthesis (TLS) polymerases that can efficiently insert nucleotides opposite a lesion at a cost of low fidelity (99). Several TLS and repair polymerases localize to the mitochondria, and they play a potential role in mtDNA replication, damage tolerance, and repair (39, 128, 151, 160, 189) (Fig. 3). A brief description of their roles in mtDNA maintenance is presented in the following paragraphs.

### PrimPol: replication repriming, TLS polymerase, and nucleotide selectivity

Human PrimPol is a monomeric DNA primase and DNA polymerase localized to both the nuclei and the mitochondria (14, 39). PrimPol contains a catalytic domain (DNA synthesis), a zinc-finger domain essential for primase activity, and two RPA-binding domains. Multiple sequence alignment of human PrimPol and putative homologs indicated that this protein is present in a broad range of unicellular and multicellular eukaryotes but likely absent in model organisms such as yeast, *Caenorhabditis elegans*, and *Drosophila melanogaster* (39).

PrimPol is a nonessential protein, but *PRIMPOL* gene silencing or ablation perturbs mtDNA replication and delays the recovery of mtDNA copy number after drug-induced depletion (39). PrimPol contributes to the maintenance of both nuclear and mitochondrial genomes mainly through its repriming activity, thus allowing the completion of DNA replication under stress conditions and at hard-to-replicate sites (14, 107, 140, 169). In addition, Torregrosa-Muñumer *et al.* (169) demonstrated both *in vivo* and *in vitro* that PrimPol can reinitiate dideoxycytidine- (ddC) and UV-stalled mtDNA replication by priming from nonconventional origins, mirroring the TLS primase function of PrimPol in the nucleus (76, 107).

Why do mitochondria need an extra primase during stress conditions? It has been proposed that alternative modes of mtDNA replication can be useful to cope with DNA damage in the mitochondria, where PrimPol could play an active role by making new primers for Polγ. Accordingly, *in vitro* assays showed that Polγ can readily use primers made by PrimPol (39, 169). Also in support of a mitochondrial function of PrimPol as part of a DNA damage tolerance pathway, this versatile enzyme is able to bypass lesions that Polγ cannot, including oxidative lesions such as 8-oxo-2′-deoxyguanosine (8-oxodG) (39, 70), apurinic, or apyrimidinic (AP-) sites (39), and UV-lesions such as cyclobutane pyrimidine dimers and 6-4-photoproducts (14, 70, 107). PrimPol acts as a TLS polymerase either by inserting a nucleotide opposite small lesions or by performing template/primer realignment based on microhomology when facing bulky “unreadable” lesions (*e.g*., UV-lesions) (94). Further, given that PrimPol shows ability to reprime downstream of G-quadruplex structures *in vitro* (140), it may also facilitate replication progression through G-quadruplexes and, possibly, other secondary structures present in the mtDNA.

Replication blocking agents, such as chain-terminating nucleoside analogs (CTNAs), can be hazardous to the very sensitive Polγ, which can incorporate, for example, ddC almost indiscriminately during mtDNA replication (36, 84). Conversely, PrimPol discriminates 187- and 233-fold better than Polγ against ddCTP and ddATP, respectively, during polymerization, implying low PrimPol-mediated toxicity with these analogs (101). In fact, cells lacking PrimPol show increased sensitivity to CTNAs commonly used in antiviral therapy, including Abacavir, Zidovudine, and Aciclovir (76). Recently, a single D114N-mutation in the catalytic domain of PrimPol was identified in an HIV-positive patient experiencing toxicity under treatment with the nucleotide analog Tenofovir (31). Notably, biochemical characterization of PrimPol^D114N^ revealed a primase-inactive protein. In addition, PrimPol-knockout cells present hypersensitivity to Tenofovir and decreased mitochondrial respiration (31). Therefore, PrimPol protects the mitochondrial genome either by not incorporating CTNAs or through its remarkable ability to restart stalled forks by making new primers for Polγ.

### Polymerase β: dRP lyase and gap filling

The DNA polymerase β is composed of two domains that are responsible for DNA synthesis and dRP lyase activities, respectively (9). Polβ is well known for its nuclear function, acting in the final steps of the base excision repair (BER) pathway by removing dRP intermediates (97) and filling gaps—mainly single-nucleotide gaps—with high fidelity (20). In the mitochondria, the gap-filling step of BER was long believed to be carried out uniquely by Polγ, which also presents dRP lyase activity (164). However, this postulate was recently challenged by the successful identification of the dedicated gap-filling Polβ in the mitochondria (128, 160).

Sykora *et al.* demonstrated that Polβ interacts with several mtDNA maintenance proteins such as TWINKLE, TFAM, SSBP1, poly(ADP-ribose) polymerase 1 (PARP1), Ligase 3 (LIG3), TDP1, and PNKP, suggesting a role in mtDNA repair (160). Mitochondrial extracts lacking Polβ have impaired BER activity on a substrate containing a single-nucleotide gap and a dRP moiety, reinforcing the role of this polymerase in short-patch mitochondrial BER (mtBER) (128). Further, Polβ-deficient mitochondria show increased mtDNA damage, mitochondrial dysfunction, and altered morphology (128, 160). Together, these recent data show that Polβ is the long-awaited missing piece of the mtBER puzzle.

### Polymerase θ: TLS polymerase, dRP lyase, and repair of double-strand DNA breaks

Human DNA polymerase θ belongs to the family A of DNA polymerases (as does Polγ) and comprises an N-terminal ATPase/helicase domain, a large central domain, and a C-terminal polymerase domain (141). Biochemical characterization of Polθ revealed DNA polymerase activity on nicked dsDNA and on a single-primed DNA template (141). Further investigation demonstrated that Polθ is an error-prone TLS polymerase that is capable of bypassing oxidative DNA lesions, thymine glycol, and AP-sites as well as extending a mismatched primer substrate (142, 143, 193). Although Polθ cannot directly insert a nucleotide opposite a cyclobutane pyrimidine dimer or a (6–4) photoproduct, it can elongate a mismatched primer containing bases placed across from these common UV-lesions (143). Polθ is proofreading-deficient and synthesizes DNA with low fidelity, thus generating single-base errors at a rate that is 10- to 100-fold higher than other family A polymerases (4). Similar to Polβ, Polθ also presents 5′-dRP lyase activity that resides in its C-terminal domain and is independent of the polymerase activity, therefore potentially contributing to short-patch BER (129).

By using an elegant siRNA screening assay in combination with a mitochondrially targeted DNA oxidizing agent (mt-Ox), Wisnovsky *et al.* reported the mitochondrial localization of Polθ, among other DNA maintenance proteins not previously associated with the organelle (189). Later studies confirmed the presence of a mitochondrial targeting sequence (MTS) in the N-terminal sequence of Polθ (190). Polθ is required for maintenance of mtDNA copy number; moreover, its mitochondrial localization is enhanced in the presence of mt-Ox, indicating a role in oxidative mtDNA damage tolerance. Accordingly, Polθ-knockout cells display lower mtDNA replication levels and decreased mitochondrial mass under oxidative stress conditions (190). Further, Polθ helps to sustain normal mitochondrial respiration and mitochondrial membrane potential in unperturbed cells, thus contributing to optimal mitochondrial function (189).

Interestingly, Polθ expression levels correlate with mtDNA point mutation frequency both in cultured cells and in tumor samples (190). Conversely, Polθ-deficient cells present decreased levels of point mutation in the mitochondrial genome (189). These data corroborate the characteristic low fidelity of TLS polymerases, suggesting that Polθ actively assists in mtDNA replication in a damaging environment by engaging in error-prone DNA synthesis. Curiously, genomic modifications such as insertions, deletions, or rearrangements were not identified in the mtDNA of cells overexpressing Polθ (190).

In the nucleus, Polθ plays a central role in double-strand break (DSB) repair *via* the microhomology-mediated end joining (MMEJ) pathway by contributing to the formation of DNA synapse, strand annealing, and DNA extension (71). Structural analysis of the helicase-like domain of Polθ showed that it exists as a tetramer, which may facilitate the alignment of broken DNA ends during MMEJ (110). Moreover, Polθ-mediated MMEJ represses the homologous recombination (HR) pathway, and its overexpression in certain HR-deficient cancers increases cell survival (17, 96). In addition, Polθ participates in immunoglobulin class-switch recombination, and it contributes to chromosomal stability and cell survival after treatment with DSB-inducing agents (194). In contrast to the established involvement of Polθ in nuclear DSB repair, the exact role of this polymerase in mtDNA maintenance remains to be elucidated.

### Polymerase ζ: TLS polymerase

Human DNA polymerase ζ is composed mainly of two subunits, the catalytic Rev3 and the accessory Rev7. The 3130 amino acid residues of human Rev3 impose a challenge for biochemical analysis; therefore, genetic studies using the yeast ortholog (1504 aa) have provided most of the information about its function. Similar to Polθ, Polζ is an error-prone and mutagenic TLS polymerase and contributes to the bypass of UV lesions and other DNA-damaging agents (89). Polζ is essential in mice, and Rev3-deficient cells show increased UV sensitivity and the accumulation of DSBs and chromosome translocations (89).

An independent study has identified an N-terminal MTS in a shorter isoform of Rev3, whose translation initiates from an alternative start site, and demonstrated the mitochondrial localization of this MTS sequence fused to GFP (151). Cellular fractionation assays also evidenced the presence of the short isoform of Rev3 in mitochondria of mouse heart and human cells (151). Currently, there is no evidence for the full-length isoform of Rev3 in the mitochondria (196). Interestingly, *REV3*^−/−^ mouse embryonic fibroblast (MEF) cells show decreased levels of the mtDNA-encoded Cox2 protein, lower Complex IV activity, decreased mitochondrial membrane potential, and increased glucose consumption, suggesting impaired mitochondrial function and a shift toward glycolysis (151). Expression levels of *Rev3* messenger RNA (mRNA) are increased in cells lacking mtDNA (ρ^0^), and in parental cells (ρ^+^) in response to OXPHOS inhibitors. Co-immunoprecipitation assays determined the interaction of Rev3 and Polγ, and reverse transcription PCR revealed increased Rev3 mRNA expression in response to a defective Polγ, indicating a role for Rev3 in mtDNA metabolism. Moreover, UV-treatment stimulates levels of *Rev3* mRNA, and in Rev3-deficient cells it causes higher expression levels of both Polγ subunits and decreased levels of mtDNA compared with Rev3-proficient cells (151). In addition, cells expressing Rev3 without the MTS region accumulate more mtDNA damage after UV exposition. Collectively, the results presented in this study indicate that Polζ contributes to mtDNA maintenance and normal mitochondrial function.

### Future perspectives on additional mitochondrial polymerases

The relatively recent discovery of additional polymerases in the mitochondria opens up new perspectives on the processes underlying mtDNA maintenance. First, an additional primase provides more possibilities for replication initiation at alternative origins and downstream of template damage. Second, the identification of TLS polymerases in the mitochondria challenges our view of mtDNA damage tolerance mechanisms. The new polymerases could potentially contribute to replication fork rescue through polymerase switching to cope with lesions that Polγ alone cannot bypass. Recent studies have demonstrated that the exceptional replication fork plasticity in the nucleus guarantees accurate DNA replication (12); likewise, the additional mitochondrial polymerases could offer plasticity during perturbed mtDNA replication. Finally, expression levels and activity of the additional polymerases—and their possible partners—could contribute to the tissue-specific differences in mtDNA maintenance mechanisms suggested by previous work (52). Unveiling the relationship between protein activity and tissue-specific mtDNA maintenance pathways will likely help us understand the clinical manifestations of mitochondrial diseases.

## Mitochondrial Nucleotide Metabolism and Incorporation of Ribonucleotides

### The mitochondrial nucleotide supply

**FIG. 4.**
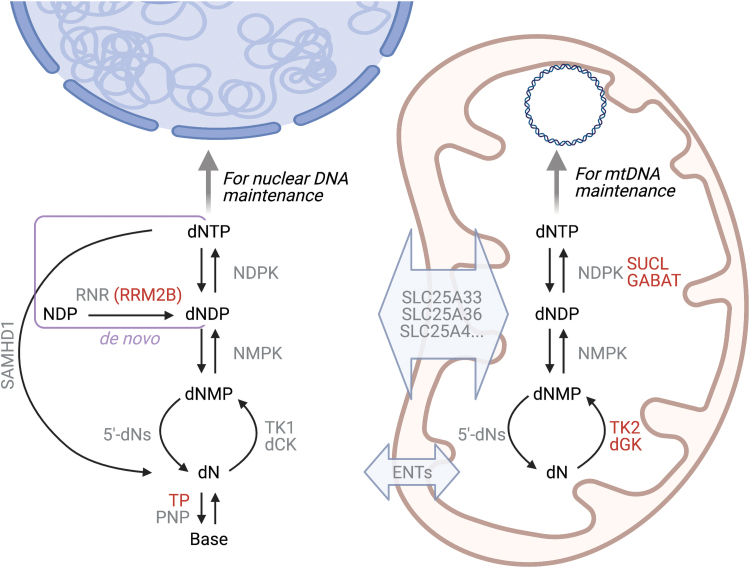
**Pathways that contribute to the mitochondrial dNTP pool.** Enzymes associated with mtDNA instability (RRM2B, TP, SUCL, GABAT, TK2, dGK) are marked in *red*. In *de novo* dNTP synthesis (*rectangle*), NDPs are reduced to dNDPs by RNR in the cytosol; the alternative small subunit, RRM2B, is required for mtDNA maintenance. The dNDPs are then phosphorylated to dNTPs by NDPK in the cytosol or in the mitochondria, where NDPK functions in complex with SUCL and the GABAT. In salvage synthesis, dNs derived from the turnover of deoxyribonucleotides (*downward arrows*) to dNMPs and further to dNs by 5′-deoxynucleotidases (5′-dNs) are phosphorylated by the sequential action of dN kinases (TK2 and dGK in the mitochondria, TK1 and dCK in the cytosol), NMPKs, and NDPKs (*upward arrows*). Note that NDPKs take part in both *de novo* and salvage synthesis. There is exchange of deoxyribonucleotides across the mitochondrial inner membrane at least *via* SLC25-family transporters, whereas dNs are exchanged *via* ENTs. In the cytosol, catabolic reactions that modulate the size of the nucleotide pool additionally include the breakdown of dNTPs to dNs by the dNTP triphosphohydrolase SAMHD1, and of dNs to their constituent bases by the nucleoside phosphorylases TP and PNP. dCK, deoxycytidine kinase; dGK, deoxyguanosine kinase; dN, deoxyribonucleoside; dNDP, deoxyribonucleoside diphosphate; dNMP, deoxyribonucleoside monophosphate; dNTP, deoxyribonucleoside triphosphate; ENTs, equilibrative nucleoside transporters; GABA, ɣ-aminobutyric acid; GABAT, GABA transaminase; NDPs, ribonucleoside diphosphates; NDPK, nucleoside diphosphate kinase; NMPK, nucleoside monophosphate kinase; PNP, purine nucleoside phosphorylase; RNR, ribonucleotide reductase; SUCL, succinate-CoA ligase; TK, thymidine kinase; TP, thymidine phosphorylase. Color images are available online.

A sufficient and balanced supply of deoxyribonucleoside triphosphates (dNTPs) is essential for mtDNA replication. In mammalian cells, the concentrations of the four dNTPs are maintained by the *de novo* and the salvage pathways. In *de novo* synthesis, dNTPs are produced by reduction of ribonucleoside diphosphates (NDPs) to deoxyribonucleoside diphosphates (dNDPs) by the enzyme ribonucleotide reductase, followed by phosphorylation to dNTPs by a nucleoside diphosphate kinase (NDPK) in either the cytosol or the mitochondria (100, 115) (Fig. 4). The *de novo* pathway produces the majority of the dNTPs found in cycling cells, and its activity peaks in S phase to provide dNTPs for nuclear DNA (nDNA) replication (115). Consequently, dNTP pools in nondividing cells, where mtDNA replication still occurs, are ∼18 times lower than in the S phase of cycling cells (47).

In contrast, salvage synthesis provides a low but constitutive level of dNTPs throughout the cell cycle. Here, already existing deoxyribonucleosides (dNs) are phosphorylated in a stepwise fashion to dNTPs. The rate-limiting step of salvage synthesis is the first phosphorylation of the dNs to yield deoxyribonucleoside monophosphates (dNMPs), which is catalyzed by thymidine kinase 2 (TK2) and deoxyguanosine kinase (dGK) in the mitochondria, whereas another pair of enzymes phosphorylates dNs in the cytosol (5). The dNMPs are then further phosphorylated to dNDPs and finally to dNTPs by the action of nucleoside monophosphate kinases (NMPKs) and NDPKs, respectively, that are found in both the cytosol and the mitochondria.

Defects in either of these dNTP synthesis pathways can severely jeopardize stable mtDNA maintenance, showing that both are of central importance for mtDNA stability (33, 173). Mutations in the RRM2B alternative small subunit of ribonucleotide reductase, the rate-limiting enzyme of the *de novo* synthesis pathway, can lead to multiple mtDNA deletions or depletion (15), as do mutations in the salvage pathway enzymes TK2 and dGK (91, 136). Also, disease-causing mutations in the SUCLA2 and SUCLG1 subunits of the citric acid cycle enzyme succinate-CoA ligase (SUCL), as well as in *ABAT* that encodes for the ɣ-aminobutyric acid (GABA) transaminase GABAT, are considered to affect the mitochondrial nucleotide supply because SUCL and GABAT form a complex with the mitochondrial NDPK isoform and are required for its activity (13, 33, 78). Other defects in nucleotide metabolism that cause mtDNA instability include mutations in MPV17, a channel-forming protein of the inner mitochondrial membrane with an as-yet unknown function (25), and those in thymidine phosphorylase (TP), a cytosolic enzyme that breaks down the nucleosides thymidine (T) and deoxyuridine (dU). TP deficiency leads to imbalanced mitochondrial dNTP pools and underlies mitochondrial neurogastrointestinal encephalomyopathy (MNGIE) with mtDNA deletions and depletion (114, 157). The multitude of enzyme defects that lead to pathological mtDNA changes highlights the central importance of nucleotide metabolism for stable maintenance of the mitochondrial genome.

### Ribonucleotide incorporation into mtDNA

rNMPs are the most common “lesions” or noncanonical nucleotides in mtDNA. Estimates of rNMP quantity in mammalian mtDNA vary depending on the cell type and the method of quantification but range from 2 to 54 rNMPs per double-stranded 16.6 kb mtDNA molecule (181). The majority of these rNMPs are not residues of unremoved replication primers or intermediates but rather single rNMPs incorporated during mtDNA replication or repair, as they appear randomly distributed over the mitochondrial genome without any detectable strand bias (10, 37, 105, 130, 183). Despite their close similarity to deoxyribonucleotides, rNMPs can threaten DNA stability due to their reactive 2′-hydroxyl group that can attack the sugar-phosphate backbone, increasing the risk of strand breaks by several orders of magnitude *in vitro* (83). Moreover, the presence of even a single rNMP can alter the elasticity and local structure of the DNA double-helix, potentially disturbing DNA–protein interactions (181).

The occasional incorporation of single rNMPs during DNA replication or repair is a consequence of the fact that free ribonucleoside triphosphates (rNTPs) are 3–100 times more abundant in the cell than the corresponding dNTPs. Therefore, even the high ability of replicative DNA polymerases to discriminate against rNTPs is insufficient to entirely prevent their insertion into DNA (44, 65, 112). This is particularly relevant for replication of mtDNA that is not restricted to the S phase of the cell cycle and thereby also occurs under conditions of high rNTP/dNTP ratios (181). Polγ incorporates fewer rNMPs than its nuclear counterparts, so the presence of rNMPs in mtDNA is not attributable to unusually frequent insertion of rNMPs but rather to the lack of efficient mechanisms to remove them.

In general, the proofreading activity of DNA polymerases is inefficient at repairing incorporated rNMPs, and Polγ is no exception to this rule (37). In the nucleus, the far majority of rNMP removal is carried out by a dedicated pathway called ribonucleotide excision repair (RER) that is initiated by the enzyme RNase H2 and additionally requires a DNA polymerase, FEN1 or another nuclease, and DNA ligase 1 (32, 135, 156). RNase H2 is absent from the mitochondria of both yeast and mammalian cells, whereby the RER pathway for efficient rNMP repair is unavailable in the mitochondrial compartment and rNMPs persist in mature mtDNA (130, 182, 183).

In addition to RER, the nuclear compartment contains an alternative pathway for rNMP removal that is mediated by Topoisomerase 1 (Top1) (75, 155). This pathway is clearly less efficient at removing rNMPs than RER, and therefore only significantly contributes to rNMP removal in the absence of RER activity (188). Although Top1-mediated removal of rNMPs can be error-free, it results in the formation of 2–5 nt deletions when it occurs at repetitive sequences, thus adding to genomic instability (75, 155, 188). It is at present unclear as to whether this alternative pathway functions in mammalian mitochondria that instead of Top1 contain a mitochondria-specific homolog, Top1mt (195). Because these two type 1B topoisomerases utilize the same reaction mechanism and share high sequence identity, theoretically Top1mt may be able to remove rNMPs. However, based on the absence of the characteristic 2–5 nt deletions at repetitive sequences despite the constant presence of rNMPs in mtDNA, the activities of Top1mt and Top1 on rNMP-containing DNA may differ.

In line with the lack of efficient rNMP removal in the mitochondria, several lines of evidence indicate that the rNMP content of mtDNA is largely determined by cellular rNTP/dNTP ratios. For example, post-mitotic tissues where dNTP levels are low contain more rNMPs per mtDNA molecule than do actively dividing cells such as cultured cells or embryos (105, 183). Further, the rNMP content in mtDNA varies between mouse tissues and correlates with the rNTP/dNTP ratio in the tissue (183). The most frequently incorporated rNMP in the mtDNA of the mouse tissues analyzed so far (liver, heart, brain) is rAMP, which is in accordance with the high levels of ATP and the consequently high rATP/dATP ratios that prevail in tissues (105, 183).

In mouse tissues just as in cultured cells, mutations that affect whole-cell and/or mitochondrial nucleotide levels alter the number and identity of rNMPs incorporated in mtDNA (10, 105, 183), leading to a discussion on the contribution of rNMPs to the pathogenic mtDNA depletion and/or deletions seen in the mitochondrial diseases involving defects in the dNTP supply. Although increased rNMP content is expected to result in mtDNA instability, it has not been unequivocally shown that the depletion or deletions observed in, for example, TK2, dGK, or MPV17 mutant cells are directly due to altered rNMPs and not a consequence of insufficient dNTP pools *per se*. Further work is, thus, needed to separate the effect of disturbed mitochondrial nucleotide pools from those of altered rNMP content.

Could rNMPs have a positive role in mtDNA metabolism? In the nucleus, the transient presence of rNMPs in newly synthesized DNA has been suggested to aid mismatch repair (MMR) (41, 87), and nicking at rNMPs may help relieve torsional stress (19). Whether the physiological level of rNMPs plays a role in mtDNA maintenance is currently unclear. Their presence is in any case not critical, as a significant decrease in mtDNA rNMP content, reached by increasing whole-body dNTP pools by ablating the SAMHD1 dNTP triphosphohydrolase, did not significantly impact mtDNA integrity or mouse lifespan (183). The jury is, therefore, still out regarding ribonucleotides in mtDNA.

## Topoisomerases: The Balance Between Topological Stress and Protein-DNA Crosslinks

Replication and transcription generate torsional stress, with positive supercoils forming ahead and negative supercoils behind the replisome or transcription complex. Without relaxation, the accumulation of these supercoils would arrest these processes, leading to genomic instability. Topoisomerases are a family of enzymes that modulate the topological structure of DNA by relieving positive and/or negative supercoils. They typically facilitate the access of DNA-binding complexes to the DNA and regulate the balance between replication and transcription. Topoisomerases also facilitate the unlinking of parental and nascent DNA molecules after completion of replication (174). All topoisomerases use a catalytic tyrosine to promote the transient cleavage of one (type I topoisomerases) or two (type II topoisomerases) DNA strands, forming a transient covalent complex called the topoisomerase cleavage complex (Top-cc) with the DNA. Once strand passage through the incision has resolved the topological strain on the DNA, the reaction is completed by ligating the DNA ends, releasing the enzyme from its substrate (174).

Mammalian mitochondria have been reported to contain at least four topoisomerases: the type I enzymes Top1mt and Top3α, and the type II Top2α and Top2β (86, 180, 195, 197). Of these, Top1mt is the only exclusively mitochondrial topoisomerase, whereas the other three are distributed in both the nucleus and the mitochondria. Top1mt is targeted to the mitochondria *via* an N-terminal MTS that is removed on import. Beyond the N-terminus, Top1mt shares high similarity with the nuclear Top1 (195). Top1mt relaxes negative mtDNA supercoils and is implicated in regulating transcription (152). Although it is not essential for mtDNA maintenance under normal conditions, Top1mt is required for recovery from severe mtDNA depletion, and has additionally been suggested to regulate mitochondrial translation (7, 30, 72, 197).

The two topoisomerase 2 isoforms, Top2α and Top2β, are encoded by separate genes (6) and differ in expression pattern, with Top2α mainly expressed in proliferating cells, whereas Top2β is more widely expressed even in post-mitotic cells (172, 184). No MTS has been identified in either isoform, but both have been detected in mitochondrial extracts, although not in all studies (50, 86, 111, 197). Neither Top2 isoform is essential for mtDNA maintenance, but Top2β is needed to relieve positive mtDNA supercoils and has been suggested to modulate mtDNA replication initiation (50, 111). Accumulating evidence suggests partial redundancy of Top1mt and the Top2 isoforms in mtDNA maintenance, because the chemical inhibition of Top2 activity in Top1mt^−/−^ mice leads to cardiotoxicity and death (73).

In contrast, the fourth mitochondrial topoisomerase, Top3α, is essential for mtDNA stability. Compound heterozygous mutations in Top3α have been reported to impair mtDNA separation after replication, leading to PEO characterized by mtDNA deletions in the muscle (111). In HeLa cells, Top3α and Top1mt were found to play synergistic roles in mtDNA replication, whereas only Top3α could separate mtDNA hemicatenanes after completion of replication (111).

Although the action of topoisomerases is beneficial for many mtDNA transactions, it can also threaten genome stability if the reaction is prevented from completing and re-sealing the DNA. Accordingly, topoisomerase poisons such as doxorubicin, etoposide, and quinolone antibiotics that trap topoisomerases in the Top-cc form on the DNA give rise to strand breaks (174). Further, these persistent protein–DNA adducts prevent the passage of replication and transcription complexes, ultimately leading to cell death if left unrepaired (127). The cytotoxic properties of topoisomerase poisons that mainly target rapidly dividing cells are exploited, for example, in anticancer or antibacterial treatment. However, some of the unwanted side effects of, for example, doxorubicin or fluoroquinolone treatment may be at least partly ascribed to mitochondrial dysfunction and the poisoning of mitochondrial topoisomerase activities, required even in postmitotic cells (43, 50, 73).

In addition to chemical poisoning of topoisomerases, persistent topoisomerase-DNA adducts can be formed at many DNA alterations, including incorporated rNMPs, AP-sites, mismatches, and oxidized nucleotides (43, 127). To protect the genome from the genotoxic effects of persistent Top-ccs, cells contain repair pathways that are capable of resolving them (127). Specifically, the tyrosyl-DNA phosphodiesterases Tdp1 and Tdp2 resolve Top1-ccs and Top2-ccs, respectively (67). Both proteins are also located in the mitochondrial compartment (55). Tdp1 has been reported to remove Top1mt-DNA adducts and promote mtDNA transcription (23). A mutation in *TDP1* leads to spinocerebellar ataxia with axonal neuropathy (SCAN1), a rare disorder that affects non-replicating neuronal cells (167). It is currently unclear whether the role of Tdp1 in resolving Top1mt-ccs contributes to the pathology of this disease, given that Tdp1 also repairs other types of DNA damage both in the nucleus and the mitochondria (179).

## Oxidative Stress and mtDNA Metabolism After Damage

Oxygen is a major source of damage to macromolecules such as the DNA. Most of the reactive oxygen species (ROS) that attack the mitochondrial and nuclear genomes are generated by electron leakage from the ETC, but they can also derive from other enzymatic processes or from exogenous agents such as ionizing and UV radiation, various toxins or drugs (158). Oxidative damage to DNA mainly leads to base modifications, some of which, if left unrepaired, can interfere with replication or impair proper base pairing. In addition to base damage, ROS can generate single- or double-strand DNA breaks, DNA-protein crosslinks, or inter-strand crosslinks (199). In this section, we review the mitochondrial repair of base modifications, whereas DNA strand breaks and DNA–protein crosslinks are discussed in subsequent sections.

### Mitochondrial repair of base modifications

**FIG. 5.**
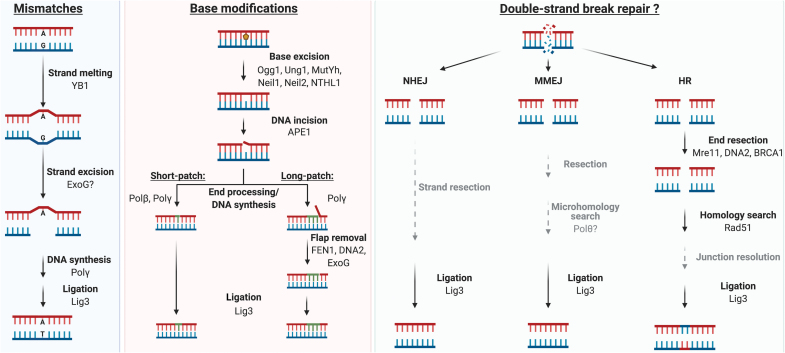
**Schematic representation of putative mtDNA repair pathways.** For the MMR (*left panel*), YB-1 is the only mitochondrial MMR-specific protein identified. The ExoG nuclease is found in mitochondria, but its role in MMR still lacks some evidence. BER (*middle panel*) responds to small base modifications, and proteins for all steps of this pathway are found in mitochondria. A range of DNA glycosylases with distinct substrate specificities can excise the damaged base; some glycosylases such as OGG1 can also incise the DNA backbone to allow excision of the AP-site. DNA synthesis occurs after end processing (details not shown). Long-patch BER involves nucleases such as FEN1, DNA2, or ExoG to remove the flap generated during strand displacement synthesis. DSB repair (*right panel*) has not been conclusively reported in mitochondria, but in the nucleus it occurs *via* HR, NHEJ, or its sub-pathway, MMEJ. Some factors of these DSB repair pathways, such as Polθ that may act in MMEJ or the HR factor Rad51, and end resection proteins Mre11, DNA2, and BRCA1 are found in mitochondria, although their roles in this organelle remain elusive. Intermediate steps that lack evidence are in *gray*. In all repair pathways, LIG3 is believed to catalyze the ligation step. HR, homologous recombination; LIG3, ligase 3; MMR, mismatch repair; NHEJ, non-homologous end joining; OGG1, 8-oxoguanine glycosylase. Color images are available online.

Base modifications and AP-sites are primarily repaired *via* BER, which was the first DNA repair pathway identified in the mitochondria and, consequently, the best understood. Briefly, BER involves the identification and removal of the damaged base, clearance of the resulting AP-site, end processing, gap filling, and ligation (Fig. 5) (178). The recognition and excision of the damaged base is carried out by specialized DNA glycosylases that cleave the N-glycosidic bond connecting the damaged base to the deoxyribose sugar, generating an AP-site. The AP-site is processed by the APE1 protein, which cuts the sugar phosphate backbone 5′ of the AP-site to produce a 3′-hydroxyl end that can be extended by a DNA polymerase (102). However, the remaining 5′-dRP residue needs to be eliminated, either by a dRP lyase activity (*e.g*., by Polβ or Polγ) in the so-called short-patch BER, or *via* strand displacement followed by exonucleolytic removal of the flap in long-patch BER. Both branches of the pathway end with ligation by LIG3 (11, 199).

The DNA glycosylases that catalyze the initial step of BER are specific to particular types of base damage, and numerous glycosylases with different but partly overlapping substrate specificities have been described in mammalian mitochondria (149). These include UNG1 and MUTYH that play a role in the repair of oxidative damage of mtDNA (22, 108). The 8-oxoguanine glycosylase (OGG1) may be translated from several alternatively spliced mRNA isoforms, resulting in proteins with mitochondrial and nuclear targeting sequences (153). The mitochondrial isoform of OGG1 is responsible for the 8-oxodG-removing activity in mouse liver mitochondrial extracts, and *OGG1*^−/−^ mice accumulate 8-oxodG in their mtDNA (153). However, the presence of OGG1 in mitochondria is not required for proper mitochondrial function or mtDNA maintenance (66, 159). NEIL1 and NEIL2 have been located in the mitochondria of cranial neural crest cells and protect mtDNA against oxidative stress during differentiation (49). Also, the NTHL1 DNA glycosylase contains nuclear and mitochondrial localization signals, and its disruption in mouse embryonic cells shows deficiency in thymine glycol metabolism (166).

For the AP endonuclease activity, mammalian cells possess the APE1 protein, which is found in both the nucleus and the mitochondria (102). APE1 is believed to be the primary source of mitochondrial AP endonuclease activity (3), although a second AP endonuclease, APE2, has also been reported in mitochondria of HeLa cells (171). As discussed in the section on Additional Polymerases Safeguard the Mitochondrial Genome, mitochondria contain at least two polymerases, Polβ and Polγ, that may be used in BER. In addition, proteins required for long-patch BER, such as the flap endonuclease FEN1 (63, 68), the endonuclease ExoG, and the helicase DNA2 (198), have been implied or localized in the organelle.

The main ligase in mtDNA metabolism, including BER, is LIG3. LIG3 is dually localized to both nuclei and mitochondria, but it is only essential for the maintenance of the mitochondrial genome (38, 150). Loss of LIG3 results in decreased mtDNA copy number and mitochondrial dysfunction (38), and the protein has been found to interact with other mtDNA metabolism proteins such as PARP1 (134), Polβ (56, 57), and NEIL1 (187). Mammalian mitochondria thereby possess all the proteins that are necessary for BER.

### MMR and nucleotide excision repair in the oxidative stress response

Although oxidative mtDNA damage is generally considered to be repaired *via* BER, additional proteins traditionally involved in other repair pathways have been found associated with the oxidative stress response. One example is the YB-1 protein, a potential factor of mitochondrial MMR, that can interact with BER enzymes such as Polβ, NEIL2, and LIG3 (26). How MMR functions in the mitochondria is still somewhat unclear. Although mitochondria appear to be able to repair G:T and G:G mismatches, key factors required for nuclear MMR are missing in mitochondria, and the MMR activity in mitochondrial extracts does not require the nuclear MMR protein MSH2 (95, 154). Therefore, mt-MMR may proceed by a distinct mechanism likely involving the mismatch-binding protein YB-1, the knockdown of which decreases MMR activity in HeLa mitochondrial extracts (154).

Proteins involved in nuclear nucleotide excision repair (NER) have also been implicated in mitochondrial oxidative stress response. Mitochondria are believed to lack a complete NER pathway that is responsible for removing bulky lesions in nDNA (120), but some components of the pathway have been identified in the organelle (64). NER is subdivided into transcription-coupled (TC-) and global genome (GG-) repair, of which the former is dependent on the CSB and CSA proteins (93). Both CSB and CSA are present in mitochondria, although at low levels (64). However, induction of oxidative stress by hydrogen peroxide (H_2_O_2_) or menadione treatment increases the mitochondrial levels of the two proteins (2, 64). Lack of CSB sensitizes cells to ETC inhibition, increases levels of mtDNA damage and 8-oxodG, and causes alterations in ETC complex organization (118). Further, cells from CSA- and CSB-deficient mutant mice show an increased mtDNA mutation rate (64). Interestingly, CSA and CSB have also been demonstrated to directly interact with SSBP1 and mitochondrial OGG1 after exposure to H_2_O_2_, implicating them in the mitochondrial oxidative stress response (64). Finally, mitochondrial extracts from CSB-deficient CS1AN cells exhibit decreased incision of 8-oxodG, implying that CSB may modulate mitochondrial BER activity (2). The effect of CSB on mitochondria is further underscored by the fact that CSB defects cause Cockayne syndrome, an early onset disease involving progeria and mitochondrial dysfunction (138).

Despite the connections to mitochondrial function and mtDNA stability, the mechanism of CSB action in the mitochondria remains unclear. CSB interacts with components of several DNA repair pathways, including PARP1, which uses NAD^+^ to catalyze poly(ADP-ribosylation) of target proteins (1). PARP1 has been suggested to control mtDNA integrity (62, 134), and besides CSB, interacts with other important mtDNA metabolism proteins, such as Polγ, APE1, LIG3, and ExoG (163). Given the interaction of CSB and PARP1, the effect of CSB on mitochondrial function and mtDNA stability may involve PARP1.

GG-NER is the branch that canonically repairs UV-induced damage to DNA. Although there is no evidence of UV-damage processing in the mitochondria, a single report demonstrated localization of the crucial GG-NER factor XPD at the inner mitochondrial membrane and showed it to increase under oxidative stress (85). Further, loss of XPD activity increased the production of mitochondrial ROS, and decreased levels of XPD delayed mtDNA repair after oxidative insults. Interestingly, silencing *XPD* increased the frequency of the common mtDNA deletion after treatment with H_2_O_2_ (85), whereas XPD deficiency during long-term exposure to non-lethal doses of UV-radiation did not (64). As many other repair proteins, XPD may, therefore, be carrying out non-canonical functions in the mitochondria.

Another crucial protein of canonical GG-NER is XPC (109), the knockdown of which leads to a shift from OXPHOS to glycolysis, together with an increase in the levels of 8-oxodG in the nuclear and mitochondrial genomes (131). XPC mutant cells presented problems in the ETC complex I and upregulation of complex II activity, leading to increased mitochondrial ROS (104). Curiously, XPC was not detected in the mitochondria (104), suggesting that nDNA repair factors and signaling can trigger imbalances in mitochondrial metabolism and affect mtDNA stability.

The effects of nDNA instability may also be mediated to the mtDNA through changes to the expression or localization of shared DNA repair factors or other nuclear-encoded mitochondrial proteins. Reversely, mtDNA instability may impact nDNA *via* altered levels of ROS, various coenzymes (ATP, NAD^+^), and metabolites such as succinate or fumarate that can influence, for example, epigenetic signatures on nDNA, among other things (106). A recent seminal study reported nDNA replication stress in induced pluripotent stem cells and MEFs with mtDNA replication defects due to an exonuclease-deficient Polγ, and it suggested this to be due to depletion of cellular dNTP pools by the mutant polymerase (48). In contrast, no dNTP pool alterations were found in mouse embryos harboring exonuclease-deficient Polγ (145). Therefore, further work is required to address the intriguing possibility of dNTPs as mediators of mito-nuclear communication. MtDNA stress may also be signaled *via* cytosolic leakage of mitochondrial nucleic acids, as discussed in the MtDNA Release and Inflammation section.

## MtDNA Strand Breaks

### Repair of mtDNA strand breaks

DNA strand breaks can arise from exogenous and endogenous sources. Single-strand DNA breaks (SSBs) usually arise as part of the repair process or by direct action of damaging agents such as oxidized bases (53, 199). On replication, a persistent SSB can generate a DSB, other sources of which include oxidative stress, ionizing radiation, and intercalating agents (148). The two major pathways for repairing DSBs in the nucleus are HR and non-homologous end joining (NHEJ) (Fig. 5).

Despite significant efforts, the presence of HR in mammalian mitochondria has not been unambiguously demonstrated and remains a subject of intense debate. If present, HR does not appear to be the major response to mtDNA DSBs. However, in a rare example of paternal mtDNA inheritance, recombination between muscle maternal and paternal mtDNA molecules has been reported (79), suggesting that recombination may take place at least in some tissues or under certain conditions. Several HR-related proteins have been reported in the mitochondria (24), and the main HR protein Rad51 has been described to promote mtDNA synthesis under replicative stress (137). However, a Rad52-like protein, necessary for the correct function of Rad51, and a mitochondrial resolvase are still missing (199). Interestingly, the mitochondrial transcription factor A TFAM shows high affinity for four-way junctions (116), and TWINKLE has been reported to catalyze strand exchange reactions (144). Mitochondria may thereby contain most of the core activities required for HR, although it is unclear whether the factors actually carry out HR in mitochondria to any appreciable extent.

The relatively frequent occurrence of large mtDNA deletions and deletion-associated mitochondrial diseases could be explained by error-prone DSB repair through an NHEJ-like mechanism (54). Because most mtDNA deletions are flanked by short microhomologies, it is interesting that an alternative form of NHEJ called MMEJ has been proposed to operate in the mitochondria. MMEJ has been confirmed in rat mitochondria, and it is dependent on FEN1, CtIP, Mre11, and PARP1 but independent of Ku70 and Ku80 (165). However, mtDNA deletion formation may also be explained through replication-dependent mechanisms such as copy-choice recombination, and recent evidence supports the ability of recombinant Polγ to generate deletions in a reconstituted *in vitro* system using a DNA template containing the direct repeats that flank the common deletion (117, 123).

In summary, further work is required to determine whether some form of DSB repair occurs in mammalian mitochondria, and what the capacity of repair is in comparison to the degradation of damaged mtDNA molecules (discussed below).

### Unrepaired mtDNA molecules

**FIG. 6.**
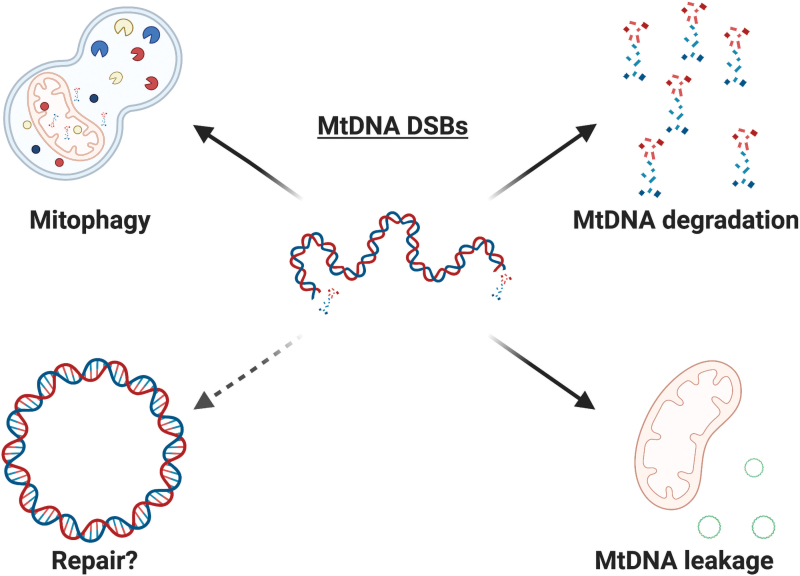
**Potential outcomes of mtDNA DSBs.** Mitochondria bearing damaged mtDNA can be recycled through mitophagy, or the damaged mtDNA molecules can be degraded. On damage, mtDNA can leak into the cytosol and elicit an immune response. Although DNA repair proteins are involved in the response to mtDNA DSBs, there is no conclusive evidence that DSB repair is occurring in mammalian mitochondria (*dashed arrow*). Color images are available online.

Damaged mtDNA molecules usually only represent a small fraction of mtDNAs in a human cell, and one process limiting their accumulation may involve the clearance of the damaged mitochondrial pool by mitophagy (82) (Fig. 6). The effects of this process are still under research, but repair proteins such as CSB have been implicated in its control. In the absence of CSB, mitophagy is downregulated and defective mitochondria accumulate in the cell, a phenotype that can be partially reversed by the use of the autophagy stimulator rapamycin (139). However, the link between mtDNA damage and mitophagy remains incompletely understood.

Another process limiting the accumulation of damaged mtDNA is the efficient degradation of damaged molecules, which occurs at least in response to overwhelming levels of mtDNA damage such as those inflicted by mitochondrially targeted restriction enzymes (103). Accordingly, a rapid decrease in mtDNA copy number and in mtDNA molecules harboring DSBs can be observed within hours after induction of mtDNA DSBs (103). The degradation has been reported to involve Polγ, TWINKLE, and the nuclease MGME1, whereas the participation of other nucleases has been excluded (103, 122). mtDNA degradation has also been observed after induction of distinct types of mtDNA damage (147). Interestingly, transient induction of low levels of DSBs in mice gave rise to mtDNA deletions rather than depletion (117). In theory, the frequency of damage may therefore determine whether the outcome of mtDNA DSBs is degradation-driven depletion or deletion formation.

### FANC proteins in mtDNA metabolism

Fanconi anemia (FA) is an autosomal recessive inherited disorder involving increased cancer susceptibility, infertility, aging, pancytopenia, and developmental abnormalities (18). It is caused by biallelic mutations in genes of the FA pathway that intersects with many other repair proteins to repair interstrand crosslinks (51). Interestingly, FA symptoms include an increased inflammatory response, mitochondrial dysfunction, and the downregulation of mitochondrial genes (119). Recently, a subset of FA factors—BRCA2 (FANCD1), RAD51C (FANCO), SLX4, and FANCD2—were demonstrated to be necessary for the protection of stalled mtDNA replication forks on oxidative damage (88). In the absence of fork protection, the nascent mtDNA was shown to be degraded by the Mre11 nuclease, leading to leakage of mtDNA into the cytosol and inflammation. We next discuss the role of mtDNA in triggering the innate immune response.

## MtDNA Release and Inflammation

**FIG. 7.**
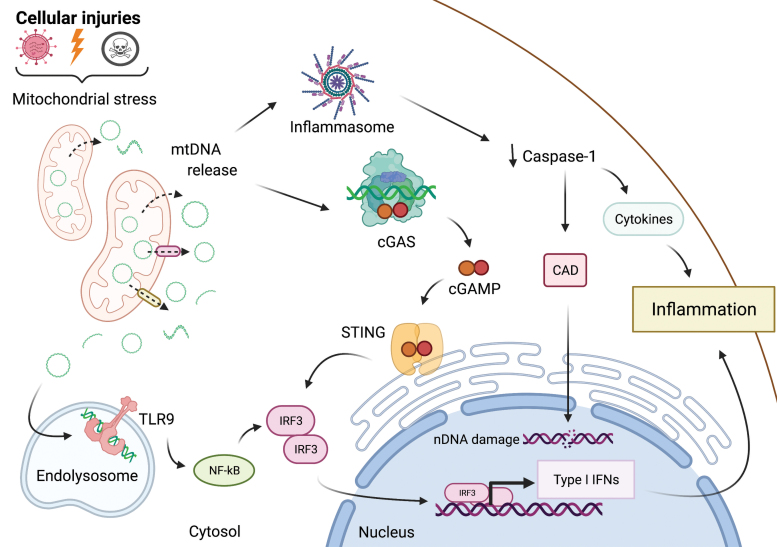
**MtDNA release and pro-inflammatory signaling.** mtDNA can trigger pro-inflammatory signaling pathways *via* cytosolic cGAS-STING and the NLRP3-inflammasome, or by endolysosomal TLR9. Cellular injuries such as infection, irradiation, or toxins trigger mitochondrial stress, resulting in the release of mtDNA into the cytosol. The NLRP3 inflammasome may be activated by mtDNA, resulting in caspase-1-mediated cytokine secretion and inflammation. In minority MOMP, a limited activation of caspase 1 results in nDNA damage induced by the CAD. cGAS recognizes cytosolic mtDNA and catalyzes the production of 2′3 ′-cGAMP, which binds and activates ER-localized STING leading to activation of TBK1. Active TBK1 phosphorylates the transcription factor IRF3, initiating an IFN I response. mtDNA in the endolysosome binds TLR9, eliciting NF-κB dependent pro-inflammatory signaling. CAD, caspase-activated DNase; cGAMP, cyclic GMP-AMP; cGAS, cyclic GMP-AMP synthase; ER, endoplasmic reticulum; IFN I, type I interferon; IRF3, interferon response factor 3; MOMP, mitochondrial outer membrane permeabilization; nDNA, nuclear DNA; NLRP3, NOD, LRR and pyrin domain-containing protein 3; STING, stimulator of interferon genes; TBK1, TANK-binding kinase 1; TLR9, toll-like receptor 9. Color images are available online.

Recent work has demonstrated that mtDNA can act as an activator of the innate immune system (133, 186, 192). Since mitochondria have their origin in endosymbiont bacteria, mtDNA is recognized as “foreign” by the cell, with some studies relating the different methylation patterns of mtDNA *versus* nDNA as a factor contributing to its immunostimulatory effect. Hypomethylation of CpG islands can make mtDNA similar to bacterial DNA, allowing the activation of pattern recognition receptors (PRRs) such as toll-like receptor 9 (TLR9) or cyclic GMP-AMP synthase (cGAS) (42, 133) (Fig. 7). Further, mtDNA metabolism can provide structures that potentially activate PRRs, such as DNA:RNA hybrids formed during transcription, long stretches of ssDNA and R-loops, which can be recognized in the cytosol by cGAS (92). Finally, TFAM, the protein that compacts mtDNA into nucleoid structures, has high immunostimulatory potential, reinforcing the idea that mtDNA and molecules associated with it serve as agonists of the innate immune system (60).

Various stresses such as mtDNA damage, defects in mtDNA maintenance factors (80), ROS, or microbial invasions (185) can result in the release of mtDNA into the cytosol, where it can come in contact with and trigger PRRs. The main route involved in the recognition of mtDNA in the cytosol is the cGAS-STING (stimulator of interferon genes) pathway, responsible for detecting the presence of invading DNA. On binding to dsDNA in the cytosol, cGAS produces 2′,3′-cyclic GMP-AMP (cGAMP), which acts as a second messenger to activate the receptor STING on the surface of the endoplasmic reticulum. STING activates TANK-binding kinase 1 (TBK1), which, in turn, phosphorylates the interferon response factor 3 (IRF3) transcription factor to promote the transcription of hundreds of interferon-stimulatory genes (ISGs), leading to the production of type 1 interferon, cytokines, and pro-inflammatory chemokines (27, 191). Cytosolic mtDNA can also engage a TLR9-dependent inflammatory response. TLR9, found on the cell surface and in the endolysosomal compartment, is activated by hypomethylated CpG motifs (8). How mtDNA is trafficked into the endolysosomal compartment is not totally clear. Autophagy is the most probable mechanism, but even mitochondria-derived vesicles (MDVs) can introduce mtDNA into an endocytic environment, where it could engage TLR9 (81).

In addition to the cGAS- and TLR9-mediated pathways, cytosolic mtDNA can activate inflammasomes. These multi-subunit complexes consist of a PRR, the adaptor protein ASC, and the cysteine protease caspase-1. Different inflammasome receptors include the NOD, LRR and Pyrin domain-containing protein 1 (NLRP1), NLRP3, NLR family CARD domain-containing protein 4 (NLRC4) and absent in melanoma 2 (AIM2). These receptors are activated by exogenous pathogen-associated molecular patterns (PAMPs) and endogenous damage-associated molecular patterns (DAMPs) released during necrosis or cellular stress (90). Inflammasome activation leads to caspase-1-mediated processing of the pro-interleukin-1β (pro-IL-1β) and pro-IL-18 into mature forms to stimulate inflammatory responses (186). Several studies have demonstrated that mtDNA can activate NLRP3- (146) and NLRC4- (59) inflammasomes and initiate a pro-inflammatory response.

The exact mechanism of mtDNA release into the cytosol remains unclear. Some studies suggest the involvement of the Bcl-2-family proteins BAX and BAK that are typically activated during mitochondrial apoptosis and drive rapid and complete mitochondrial outer membrane permeabilization (MOMP). After the formation of BAX/BAK pores, pro-apoptotic proteins are released and initiate the caspase cascade, culminating in cell death (98, 132). Growth of the outer membrane pores to macropores allows herniation of the inner membrane and extrusion of mtDNA (133). Usually, apoptotic caspases cleave key proteins that are involved in activation of the immune response, dampening the immune response during programmed cell death (113). However, under some conditions of sublethal stress, only a fraction of mitochondria undergo MOMP and trigger limited caspase activity that can be aborted before the point of no return. In this scenario, termed “minority MOMP,” the leakage of mtDNA into the cytosol stimulates cGAS-STING, causing inflammation and activation of the immune system (16). Therefore, rather than leading to destruction of the cell, minority MOMP leads to inflammation that is believed to have a signaling role. However, signaling *via* minority MOMP comes at a cost: even though the apoptotic signal is insufficient for caspase-3 activation, it is high enough to activate CAD (caspase-activated DNase) that attacks nDNA, jeopardizing genome integrity (58). Even pathways other than BAX/BAK may be involved in the release of mtDNA, such as the formation of mitochondrial permeability transition pore (mPTP) (40, 121), voltage-dependent anion channel (VDAC) (74), and MDVs (81). Further, some stresses such as mtDNA DSBs have been reported to trigger an immune response through the release of mitochondrial RNA, not mtDNA (168).

In conclusion, recent evidence suggests that mtDNA can be used as a signal of various genotoxic or stress conditions to trigger antimicrobial and inflammatory responses. MtDNA release plays a role in pathophysiological situations such as viral infection, and it has been speculated to contribute to the inflammation observed in neurodegenerative diseases such as Parkinson's disease (133, 186).

## Concluding Remarks

The importance of an intact mitochondrial genome has gained appreciation as being critical for the process of OXPHOS since the discovery of the first pathological mtDNA alterations in the late 1980s (54, 177). We now know that mtDNA instability in the form of deletions, point mutations, or copy number depletion causes a large number of mitochondrial diseases, and it is additionally associated with various pathophysiological states and aging (33, 161). Although the importance of faithful mtDNA maintenance is clear, we still lack knowledge of many of the molecular-level mechanisms involved in protecting this genome. Which of the many nDNA repair enzymes that have been reported to localize to the mitochondria really play a significant role there, and how do their functions in the compartment compare with their canonical ones? These questions are challenging to address with dually localized enzymes, unless the mitochondrial isoform can be specifically removed. Further complexity is introduced by the constant communication of the mitochondria with their surroundings—nDNA stress can impact mtDNA maintenance *via*, for example, shared repair factors or the nucleotide pools, and very recent findings highlight the possible role of mtDNA maintenance defects in nDNA stability [*e.g*., Hämäläinen *et al*. (48)]. In addition, damaged mtDNA molecules may sometimes be degraded rather than repaired, but a complete understanding of what regulates the balance of degradation *versus* repair remains enigmatic. Finally, the intriguing role of mtDNA itself acting as a signal of cellular or mitochondrial stress has emerged with full power, and it raises numerous as-yet unanswered questions that the mitochondrial community should address in the coming years.

## Abbreviations Used

8-oxodG: 8-oxo-2′-deoxyguanosine
AP: apurinic or apyrimidinic
BER: base excision repair
cGAMP: cyclic GMP-AMP
cGAS: cyclic GMP-AMP synthase
CAD: caspase-activated DNase
CTNA: chain-terminating nucleoside analog
dCK: deoxycytidine kinase
ddC: dideoxycytidine
dGK: deoxyguanosine kinase
dN: deoxyribonucleoside
dNDP: deoxyribonucleoside diphosphate
dNMP: deoxyribonucleoside monophosphate
dNTP: deoxyribonucleoside triphosphate
dRP: deoxyribose phosphate
DSB: double-strand break
dsDNA: double-stranded DNA
ENT: equilibrative nucleoside transporter
ER: endoplasmic reticulum
ETC: electron transport chain
FA: Fanconi anemia
GABA: ɣ-aminobutyric acid
GABAT: GABA transaminase
GG: global genome
H_2_O_2_: hydrogen peroxide
HR: homologous recombination
IFN I: type I interferon
IL: interleukin
IRF3: interferon response factor 3
LIG3: ligase 3
MDV: mitochondria-derived vesicle
MEF: mouse embryonic fibroblast
MMEJ: microhomology-mediated end joining
MMR: mismatch repair
MOMP: mitochondrial outer membrane permeabilization
mRNA: messenger RNA
mtBER: mitochondrial BER
mtDNA: mitochondrial DNA
mt-Ox: mitochondrially-targeted DNA oxidizing agent
MTS: mitochondrial targeting sequence
nDNA: nuclear DNA
NDP: ribonucleoside diphosphate
NDPK: nucleoside diphosphate kinase
NER: nucleotide excision repair
NHEJ: non-homologous end joining
NLRC4: NLR family CARD domain-containing protein 4
NLRP: NOD, LRR and Pyrin domain-containing protein
NMPK: nucleoside monophosphate kinase
OGG1: 8-oxoguanine glycosylase
O_H_: origin of Heavy-strand replication
O_L_: origin of Light-strand replication
OXPHOS: oxidative phosphorylation
PARP1: poly(ADP-ribose) polymerase 1
PEO: progressive external ophthalmoplegia
PNP: purine nucleoside phosphorylase
Polγ: polymerase γ
PRR: pattern recognition receptor
RER: ribonucleotide excision repair
rNMP: ribonucleoside monophosphate (incorporated ribonucleotide)
RNR: ribonucleotide reductase
rNTPs: ribonucleoside triphosphates
ROS: reactive oxygen species
SSB: single-strand DNA break
SUCL: succinate-CoA ligase
TBK1: TANK-binding kinase 1
Tdp: tyrosyl-DNA phosphodiesterase
TK: thymidine kinase
TLR9: toll-like receptor 9
TLS: translesion synthesis
Top1: topoisomerase 1
Top-cc: topoisomerase cleavage complex
TP: thymidine phosphorylase
UV: ultraviolet
